# Supplementary material for: Multi‐dataset identification of innovative feature genes and molecular mechanisms in keratoconus
Source: J Cell Mol Med. 2024 Sep 19;28(18):e70079. doi: 10.1111/jcmm.70079 (PMC11412914; doi:10.1111/jcmm.70079)
Supplement: Supplementary file 2 — Material S2. [file JCMM-28-e70079-s002.zip › 7.GSEA analysis/1.ARL11/KEGG_BASAL_CELL_CARCINOMA.html]

Details for gene set KEGG\_BASAL\_CELL\_CARCINOMA[GSEA]

|  || Dataset | ARL11.group.cls#h\_versus\_l |
| Phenotype | group.cls#h\_versus\_l |
| Upregulated in class | l |
| GeneSet | KEGG\_BASAL\_CELL\_CARCINOMA |
| Enrichment Score (ES) | -0.42078897 |
| Normalized Enrichment Score (NES) | -1.2506027 |
| Nominal p-value | 0.19103314 |
| FDR q-value | 1.0 |
| FWER p-Value | 0.973 |
Table: GSEA Results Summary

  

Fig 1: Enrichment plot: KEGG\_BASAL\_CELL\_CARCINOMA      
 Profile of the Running ES Score & Positions of GeneSet Members on the Rank Ordered List

  

| SYMBOL | TITLE | RANK IN GENE LIST | RANK METRIC SCORE | RUNNING ES | CORE ENRICHMENT || 1 | APC2 | na | 1889 | 0.305 | -0.0092 | No |
| 2 | WNT5A | na | 3234 | 0.230 | -0.0138 | No |
| 3 | WNT9A | na | 5395 | 0.155 | -0.0531 | No |
| 4 | FZD4 | na | 5857 | 0.141 | -0.0455 | No |
| 5 | WNT10B | na | 6703 | 0.119 | -0.0523 | No |
| 6 | SMO | na | 7558 | 0.101 | -0.0620 | No |
| 7 | PTCH1 | na | 7694 | 0.098 | -0.0513 | No |
| 8 | GLI3 | na | 7876 | 0.095 | -0.0424 | No |
| 9 | FZD1 | na | 8051 | 0.091 | -0.0338 | No |
| 10 | SUFU | na | 8482 | 0.083 | -0.0339 | No |
| 11 | WNT3A | na | 8640 | 0.080 | -0.0265 | No |
| 12 | TP53 | na | 9447 | 0.068 | -0.0396 | No |
| 13 | TCF7L2 | na | 10815 | 0.050 | -0.0717 | No |
| 14 | FZD5 | na | 11914 | 0.037 | -0.0978 | No |
| 15 | FZD6 | na | 12404 | 0.032 | -0.1071 | No |
| 16 | WNT8A | na | 12464 | 0.032 | -0.1041 | No |
| 17 | FZD3 | na | 12567 | 0.031 | -0.1025 | No |
| 18 | WNT1 | na | 12803 | 0.029 | -0.1050 | No |
| 19 | CTNNB1 | na | 14653 | 0.014 | -0.1563 | No |
| 20 | GLI2 | na | 15544 | 0.007 | -0.1809 | No |
| 21 | WNT3 | na | 16162 | 0.003 | -0.1983 | No |
| 22 | WNT10A | na | 16653 | -0.001 | -0.2123 | No |
| 23 | HHIP | na | 17366 | -0.006 | -0.2319 | No |
| 24 | TCF7 | na | 18185 | -0.012 | -0.2538 | No |
| 25 | WNT4 | na | 18621 | -0.014 | -0.2642 | No |
| 26 | AXIN2 | na | 19144 | -0.018 | -0.2766 | No |
| 27 | DVL3 | na | 19519 | -0.021 | -0.2843 | No |
| 28 | STK36 | na | 19690 | -0.022 | -0.2860 | No |
| 29 | WNT5B | na | 19914 | -0.023 | -0.2890 | No |
| 30 | FZD10 | na | 20030 | -0.024 | -0.2888 | No |
| 31 | GSK3B | na | 20728 | -0.029 | -0.3045 | No |
| 32 | DVL2 | na | 22249 | -0.042 | -0.3421 | No |
| 33 | WNT2 | na | 22576 | -0.046 | -0.3447 | No |
| 34 | DVL1 | na | 23820 | -0.060 | -0.3716 | No |
| 35 | TCF7L1 | na | 24530 | -0.070 | -0.3817 | No |
| 36 | AXIN1 | na | 24756 | -0.073 | -0.3774 | No |
| 37 | FZD9 | na | 25720 | -0.086 | -0.3924 | No |
| 38 | WNT7A | na | 26706 | -0.103 | -0.4056 | Yes |
| 39 | BMP4 | na | 27068 | -0.110 | -0.3997 | Yes |
| 40 | FZD8 | na | 27395 | -0.117 | -0.3918 | Yes |
| 41 | WNT7B | na | 27789 | -0.125 | -0.3845 | Yes |
| 42 | WNT16 | na | 28062 | -0.132 | -0.3727 | Yes |
| 43 | PTCH2 | na | 28260 | -0.138 | -0.3579 | Yes |
| 44 | FZD2 | na | 28464 | -0.142 | -0.3426 | Yes |
| 45 | APC | na | 29306 | -0.166 | -0.3422 | Yes |
| 46 | WNT6 | na | 29533 | -0.173 | -0.3230 | Yes |
| 47 | FZD7 | na | 30073 | -0.190 | -0.3103 | Yes |
| 48 | BMP2 | na | 30615 | -0.208 | -0.2950 | Yes |
| 49 | WNT9B | na | 30689 | -0.211 | -0.2659 | Yes |
| 50 | WNT8B | na | 31805 | -0.253 | -0.2605 | Yes |
| 51 | LEF1 | na | 32575 | -0.292 | -0.2393 | Yes |
| 52 | SHH | na | 33891 | -0.404 | -0.2172 | Yes |
| 53 | WNT11 | na | 33983 | -0.417 | -0.1579 | Yes |
| 54 | GLI1 | na | 34157 | -0.445 | -0.0968 | Yes |
| 55 | WNT2B | na | 34700 | -0.762 | 0.0008 | Yes |
Table: GSEA details [plain text format]

  

Fig 2: KEGG\_BASAL\_CELL\_CARCINOMA      
 Blue-Pink O' Gram in the Space of the Analyzed GeneSet

  

Fig 3: KEGG\_BASAL\_CELL\_CARCINOMA: Random ES distribution      
 Gene set null distribution of ES for **KEGG\_BASAL\_CELL\_CARCINOMA**
